# The Strategy against Iatrogenic Prematurity Due to True Umbilical Knot: From Prenatal Diagnosis Challenges to the Favorable Fetal Outcome

**DOI:** 10.3390/jcm11030818

**Published:** 2022-02-03

**Authors:** Roxana Elena Bohiltea, Valentin-Nicolae Varlas, Vlad Dima, Ana-Maria Iordache, Teodor Salmen, Bianca-Margareta Mihai, Alexia Teodora Bohiltea, Emilia Maria Vladareanu, Ioniță Ducu, Corina Grigoriu

**Affiliations:** 1 Department of Obstetrics and Gynaecology, “Carol Davila” University of Medicine and Pharmacy Bucharest, 37 Dionisie Lupu, 020021 Bucharest, Romania; corigri@gmail.com; 2 Department of Obstetrics, Gynaecology and Neonatology, Filantropia Hospital, 11-13 Ion Mihalache Blv., Sector 1, 011171 Bucharest, Romania; bmmihai@gmail.com; 3 Optospintronics Department, National Institute for Research and Development in Optoelectronics-INOE 2000, 409 Atomistilor, 077125 Magurele, Romania; 4 Department of Diabetes, Nutrition and Metabolic Diseases, “Prof. Dr. N.C.Paulescu” National Institute of Diabetes, Nutrition and Metabolic Diseases, 030167 Bucharest, Romania; teodor.salmen@gmail.com; 5 École Hôtelière de Lausanne EHL Hospitality Business School, 1000 Lausanne, Switzerland; 6 Faculty of Medicine, “Carol Davila” University of Medicine and Pharmacy Bucharest, 37 Dionisie Lupu, 020021 Bucharest, Romania; vladareanu@gmail.com; 7 Department of Obstetrics and Gynaecology, University Emergency Hospital, 169 Splaiul Independentei Bld., Sector 5, 050098 Bucharest, Romania; ionitaducu@gmail.com

**Keywords:** true umbilical knot, premature birth, 3D-HD Flow Color Doppler, prenatal diagnosis, intrauterine fetal death, umbilical cord

## Abstract

True umbilical knot (TUK), although not a commonly encountered pathology, hasan important psychological burden on the mother and obstetrician. It has an extremely low prenatal ultrasound diagnosis rate, despite its adverse perinatal outcomes when unknown. We conducted a retrospective observational analytical study on a 7-year period (2015–2021), including all pregnancies overseen by a single fetal-maternal medicine specialist for monitoring and delivery. We analyzed the prenatal detection rate and correlations between prenatal diagnosis of TUK and pregnancy outcome in terms of associated maternal and fetal factors, time and mode of delivery, fetal weight at birth, maternal level of stress, and iatrogenic prematurity. We compared our results with an electronic search of the literature to study the relationship between TUK and prematurity. We prenatally diagnosed 16 TUKs, and there were two false positives and two undiagnosed knots. All of those women had birth at term. The main finding of the review was a small number of studies that included enough cases for analysis. The prematurity rate due to TUK is 14.2%, significantly increased compared to the general population. An umbilical artery flow velocimetry notch in twin pregnancies complicated by TUK was an important ultrasonographic finding. We consider intrauterine fetal death exceptional, and the main adverse neonatal outcome is due to iatrogenic prematurity caused by maternal anxiety of knowing the prenatal diagnosis and mode of delivery. The elective method for diagnosis should be the second-trimester ultrasound scan using three-dimensional (3D) reconstruction and cesarean delivery for a good neonatal outcome. Pregnant women should be counseled to understand the implications of iatrogenic prematurity, especially respiratory distress syndrome, to ensure these infants are delivered at term.

## 1. Introduction

Umbilical cord and placental abnormalities account for approximately 30% of intrauterine deaths [1]. Umbilical knots amount to about 1.1% of cases [2]. The intrauterine fetal death rate is 4 to 10 times higher in cases of true umbilical knot [3]. The incidence of true knots is 0.3–2.1%, and advanced maternal age and male fetuses seem to be risk factors [4]. The maternal pathologies that are associated with true umbilical knots (TUKs) are chronic hypertension and gestational diabetes. The newborns are affected by preterm delivery. They also have low Apgar scores and low birth weight, apparently due to the compression of umbilical vessels causing narrowing and decreased blood flow [5].

Certain studies have hypothesized the mechanisms through which true knots could cause intrauterine death by cord occlusion. One such study [6] published in 1977 showed that flattening or dissipation of Wharton’s jelly and venous congestion and partial/complete vascular occlusion are the determinant factors that produce intrauterine fetal death in pregnancies complicated with umbilical knots. Another study focused on the blood gas values in newborns with and without true knots. The results showed no significant differences between the arterial and venous values of pH, pCO_2_ (partial pressure of CO_2_ gas), pO_2_ (partial pressure of O_2_), and HCO_3_^−^ (hydrogen carbonate) in the control group and the true knots group [7].

A recent study concluded that children born with true umbilical cord knots are not at increased risk of developing long-term neurological complications [8], but the obstetric associations include increased incidences of small-for-gestational-age infants, premature birth, need for neonatal intensive care, and intrauterine death [9,10].

Even if most ultrasound societies do not recommend screening for umbilical cord abnormalities [11,12,13,14,15], the second trimester is the best window to detect by two-dimensional ultrasound (2D-US) the entanglement of the umbilical cord. There is suspicion of the knots being strengthened if color Doppler is used, as some case studies have presented [16,17,18,19]. Confirmation is obtained by three-dimension (3D) HD-flow color Doppler [20]. A recent review article [1] proposed second-trimester screening for umbilical cord abnormalities; however, visualization of the cord insertions and trajectory becomes difficult with advancing gestational age. Other studies have highlighted the need to perform a sonographic evaluation of the cord coiling and looping, and Doppler velocimetry studies [21,22].

This paper aims to sustain the roles of Doppler ultrasound in (i) detecting the presence of true umbilical knots, (ii) evaluating the hemodynamic status of the fetal circulation, (iii) guiding the future management of the pregnancy, and (iv) preventing iatrogenic preterm birth.

The article emphasizes the rationale behind the theory that intrauterine growth restriction and intrauterine fetal demise due to umbilical knot tightening are exceptionally rare, and the most important adverse neonatal outcomes originated in iatrogenic premature birth. The psychological impact of the prenatal diagnosis of umbilical cord knot on the obstetricians and mothers, aware of high risk of intrauterine fetal death, is able to cause a premature delivery.

## 2. Materials and Methods

We conducted a retrospective observational analytical study on 7-year period (2015–2021), in the “Emergency University Hospital”, situated in Bucharest, Romania, a tertiary obstetrical center specializes in managing high-risk pregnancies. We included pregnancies addressed to a single fetal-maternal medicine specialist for monitoring and birth. We analyzed the prenatal detection rate and correlations between prenatal diagnosis of true umbilical knot and pregnancy outcome in terms of associated maternal and fetal factors, time and mode of delivery, fetal weight at birth, postnatal confirmation of ultrasound findings, maternal level of stress, and iatrogenic prematurity.

Obstetrical ultrasound assessment routinely uses 2D trans-abdominal and transvaginal grayscale, color Doppler and three-dimensional ultrasound; the investigation is performed with a Voluson E8 (GE Medical), equipped with a Ge Rab4-8-D Ultrasound Probe.

The study was conducted following the Declaration of Helsinki and approved by the Institutional Review Board of Life Memorial Hospital (protocol code 1/03.01.2022). Written informed consent has been obtained from the patients to publish this paper.

We compared our results with other studies to find the relationship between TUKs and prematurity. We performed an electronic search of the literature of the following MeSH terms: “umbilical cord knot”, “mode of birth”, “cesarean section”, “vaginal birth”, “ultrasonography”, and “prenatal diagnosis” with a data filter on the search (AND) “*premature birth*” over 10 years. The search aimed to identify clinical case series (CCS) and case reports (CR); the last updated search was performed on 30 November 2021. The review involved a search of PubMed^®^/MEDLINE databases and the Web of Science (WoS) Core Collection.

Inclusion criteria were the following: clinical case series and case reports published in the last 10 years, investigating (1) umbilical cord knot and prematurity among studies, (2) neonatal outcomes, (3) main findings on ultrasound examination. Exclusion criteria were the following: (1) other types of documents than CCS and CR, (2) articles off-topic or with insufficient information, (3) studies published before 2011.

We used the Romanian adapted version DASS-21R from the original DASS version questionnaire to quickly assess the emotional status (anxiety) of pregnant women regarding the prenatal diagnosis of TUK and the manner of birth [23,24]. A single-factor structure on the DASS-21 scale was investigated that contained 7 questions to measure the degree of maternal anxiety; the assessment of internal reliability was determined with the help of Cronbach Alpha.

Data analysis was realized using the SPSS^®^ 27.0 software (IBM^®^Corp., Armonk, NY, USA). We showed the continuous variables using the median (range), mean and standard deviation (SD) with a 95% confidence interval (CI) or count (percent, %). We calculated the specificity, sensitivity and accuracy of the prenatal diagnosis method.

## 3. Results

From January 2015 to November 2021, we registered 588 pregnancies which were monitored by ultrasound and birth assisted by the same obstetrician and fetal-maternal specialist. During this period, 16 pregnancies complicated by apparent umbilical cord knots were detected by ultrasound, but only 14 were confirmed after birth; two cases proved to be false knots (cases 10 and 13), and another two cases were prenatally undiagnosed, but postnatally found. In our group, there were 15 singleton and 1 twin pregnancies. The characteristics of the umbilical knot pregnancies are shown in Table 1 and Table S1. In the twin pregnancy (case 16), due to the spatial architecture of the cord entanglement in the third trimester, we suspected a false knot, but it was a real one.

The calculated prevalence of the true umbilical cord knot in the population group was 2.72%. The sensitivity of the umbilical cord knot detection by ultrasound was 87.5%, the specificity was 99.6%, and the accuracy was 96.9%.

**Figure 1:**
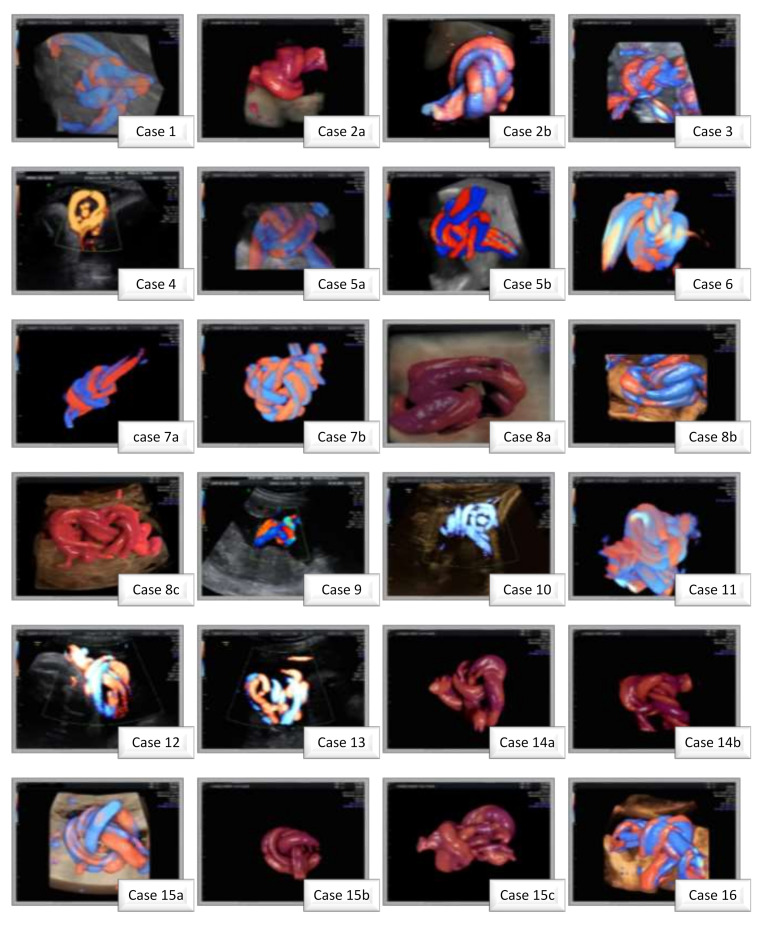
Power Doppler Ultrasound and and 3D-HD-Flow recontructed images of the prenataly diagnosed umbilical knots observed ofin the cases presented in this paper.

The median gestational age at diagnosis was 25.5 ± 6.7 weeks (range: 16–37): two cases were detected at the 16th week, two cases were detected near term, four cases were detected in the third-trimester ultrasound scan at 30 weeks, and the majority of cases were detected during the second-trimester ultrasound screening for fetal anomalies (8 cases, 57.1%). Interestingly, 7 of 14 pregnancies presented a nuchal cord, three of which were double, and the knot was complex in two cases (Figure 1).

The average maternal age was 33 (±5.06) years old; the range was 28–42. However, only three knots occurred in cases over 35 years of age. The obstetrician subjectively evaluated the level of anxiety after diagnosis and counseling. The DASS-21R scale has five levels of appreciation (normal, low, moderate, severe, and extremely severe), and in our study, we found low (37.5%), moderate (25%), and severe (37.5%) forms of anxiety. Chronbach’s alpha coefficient was 0.771, which means a good level of reliability.

Polyhydramnios was present in two cases and oligohydramnios in one case. Thirteen cases had amniotic fluid indexes in the respective normal ranges for gestational age until delivery. Gestational insulin-dependent diabetes mellitus occurred in one case. Another three babies manifested altered responses to a tolerance of glucose test performed between 24 and 28 weeks. Mild pregnancy-induced hypertension was present in the only twin pregnancy case.

Regarding fetal variables, 35.7% of the children were male, and the average birth weight was 3332.85 ± 277 g (range: 2880–3800). All newborns were delivered by cesarean section and presented normal growth and development for the gestational age at birth, and there were no intrauterine growth restriction or stillbirth cases. In addition, none of the 16 newborns (including two false-positive cases) were delivered earlier due to maternal anxiety.

In the topic search, more than 71 papers were screened for eligibility, and 13 were identified and analyzed. The main results are presented in Table 2.

## 4. Discussion

The main finding of the literature search was that, although true umbilical knot has a significant incidence and the results could be life-threatening, there are very few studies in the literature containing large numbers of cases. Instead, the vast majority are case reports and small case studies. Of the selected articles, we included 13 studies in Table 2, and the results of these studies emphasize that from 1012 pregnancies with a true umbilical knot, the prematurity rate was 14.2% (144 cases).

The only large retrospective study analyzing 85,541 deliveries, among which 867 cases of true umbilical knot included information on pregnancy outcome, was based exclusively on the postnatal diagnosis of true umbilical knot. In this very recent article published by Weissmann-Brenner et al. [35], the rate of cesarean delivery of the study group with true umbilical knots was similar to the one obtained in the control group containing pregnancies without true umbilical knots, but the rate of cesareans for acute fetal distress was significantly higher in the umbilical cord knot group, which resulted in a fetal death rate 2.5 fold higher.

In a 23-year retrospective study of 243,639 newborns, Lichtman et al. showed that 1.1% of them had TUKs. The analysis revealed more preterm births (10.5% TUK versus 6.8% non-TUK) and cesarean deliveries (17.4% TUK versus 13.5% non-TUK). However, the Apgar score <7 at 1 min in the TUK group was 7.3%, and perinatal mortality was 1.8% [8].

Regarding the delivery mode of patients with TUK, the incidence of cesarean section varies between 17.4 and 27.8%, and the induction of labor is 28.7–87% [8,36]. However, in our study, the method of birth was the cesarean section in all cases, explained by the increased anxiety of the patients, the fear of fetal death, and the obstetrician’s concern about malpractice.

Two of the three studies regarding twin pregnancies with true umbilical knots have described a notch in the umbilical artery flow velocimetry in one of the umbilical cords [8,29]; two of the four cases presented ultrasonographic cord entanglement, and the third was diagnosed prenatally with true umbilical cord knot; the latter did not have any ultrasonographic modifications [37].

However, a recent study [3] showed that excessively long cords are not associated with intrauterine fetal demise in singleton pregnancies. Compared with false knots (which are formed by the twisting of the umbilical vein around the umbilical artery), true knots show definitive compression of the cord vessels and loss of Wharton’s jelly (which may be associated in some cases with edema, slower venous drainage, and vascular congestion) even after the knot is released [7].

The diagnosis of a true knot is difficult to assess by two-dimensional (2D) ultrasound because of its three-dimensional structure (Video S1). In 2D ultrasound, three typical signs are indicators of an umbilical knot: (1) “hanging noose” [7,38]; (2) “four-leave clover”, and (3) unusual multicolor pattern of the umbilical cord [37]. Moreover, the 3D/4D Doppler can “reconstruct” the trajectory of the umbilical cord and actively identify the knot and determine the degree of constriction. A false knot can be differentiated from a true one by rotating the volume and re-visualizing the cord from a different angle [37].

Usually, true knots appear in multiparous women [4,29] and can be visualized starting from the second trimester [31]. Therefore, the blood flow indexes (PI, RI, PSV) should be evaluated by Doppler ultrasonography after 20 weeks of pregnancy [4]. However, even using Doppler imaging and Doppler velocimetry, false-negative results occur because: (1) the entirety of the cord cannot be seen or assessed, and (2) some knots can be obscured from sight [39].

The next step in visualizing the umbilical cord insertions and trajectory is virtual reality, which offers more precise measurement and more “real” visualization of the complex structures. Such a study has been published to analyze the umbilical cord and vitelline duct, representing the first step in this direction [40]. In addition, a study published in 2014 by Ugurlucan and Yuksel [41] sustains the feasibility of scanning the entire umbilical cord during the second-trimester scan.

Our study included 14 cases of diagnosed TUK and two cases of undiagnosed umbilical knots from 588 scanned pregnancies, but none of the true knots presented signs of intrauterine or postpartum tightening. More than this, multiparity and advanced maternal age were less represented in our group (5 and 3 out of 14 cases) in contrast to published data which associate true umbilical knots with multiparity, advanced maternal age, male fetus, excessively long umbilical cord, and intrauterine fetal death [16].

Prenatal ultrasound diagnosis is difficult because it involves performing real-time 2D and 3D ultrasound, a method of examination that is not widely used. The latter can increase the accuracy of the diagnosis and differential diagnosis with respect to the false cord knot. Our data supporting the results obtained by Rodrigues et al. [26]. In 1995, Sepulveda et al. showed that antenatal diagnosis of TUK in second and third-trimester ultrasound scans is easy to miss, all cases being diagnosed at birth [42]. Gembruch and Baschat showed for the first time transient changes in the umbilical cord knot secondary to the stenotic effect on umbilical vein flow through the use of color and power Doppler [43]. The use of power Doppler 3D ultrasound in TUK prenatal diagnosis highlighted its vascular spatial pattern with a birth confirmation rate ranging from 62.5 to 96.4% [36,44]. The role of 4D ultrasound in the differential diagnosis of true cord knots was also studied without demonstrating superiority over 3D evaluation [45].

Furthermore, Sherer et al. highlighted that their association of true umbilical knot with a nuchal cord node might increase the risk of adverse perinatal outcomes. All three of their cases were born by cesarean section [33].

According to Hershkovitz’s study, TUK may be associated with an increased incidence of cesarean section due to uncertain fetal status during labor, or with obstetric factors (gestational diabetes, SGA, polyhydramnios, long umbilical cord, and genetic amniocentesis) [46]. Guzikowski showed that only 40% were diagnosed prenatally. Improving the prenatal diagnosis of TUK would help reduce the incidence of uncertain fetal status [4]. Hasbun, using 3D Doppler ultrasound, identified TUK prenatally in 62.5% of cases [44].

**Figure 2:**
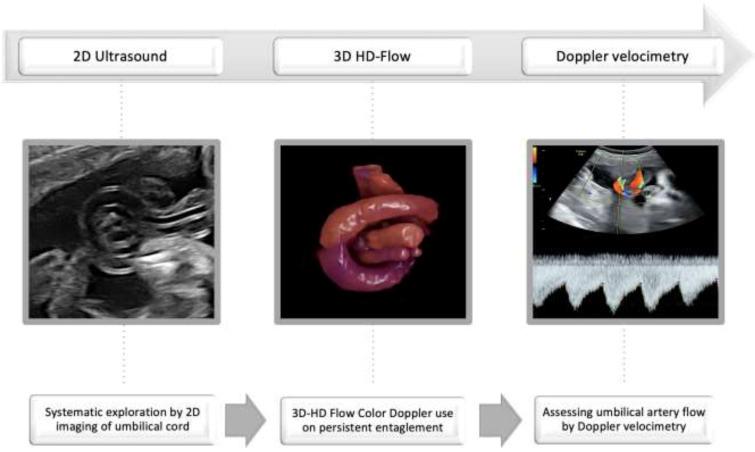
The strategy of prenatal diagnosis and monitoring true umbilical knot to term.

Considering our experience and published data, we developed an algorithm for increasing the umbilical cord knot detection rate (Figure 2).

A systematic look after umbilical cord entanglement during the second trimester 2D ultrasound screening for fetal anomalies should be followed by 3D-HD-flow color Doppler use, or by checking the persistence entanglement after fetal movement induction or after 1–2 weeks until reevaluation, if incertitude persists and 3D imaging is unavailable. Doppler velocimetry of the umbilical artery should be the monitoring standard for the last trimester, and repeated weekly in the last month until term delivery.

Technological progress, increasing the number of maternal and fetal medicine specialists using the 3D technique, and paying special attention to umbilical cord pathology will increase the rate of prenatal detection, contributing to decreased perinatal morbidity and mortality.

Early diagnosis of umbilical cord knot increases the anxiety of both the mother and the obstetrician, carrying the risk of inducing a premature birth in many situations, as is easily seen in our reviewed data: four articles, including our previous report, affirmed iatrogenic prematurity due to maternal anxiety [31]. In contrast, antepartum non-recognition of this pathology is often associated with adverse perinatal outcomes. Interestingly, the only case of premature delivery occurred in the twin pregnancy and was imposed by velamentous insertion of umbilical cords and premature preterm rupture of membranes, without any pressure due to anxiety from the parents. We are also aware of the pressure on the obstetrician both from the knowledge that intrauterine death is four to ten times higher in TUK cases and from the parents’ concern; they are often forced to practice defensive medicine and deliver the babies earlier. The rate of preterm birth in our study was 14.2%, though in two others the rates were only 10.5 and 10.9% [8,35].

The role of this study is not only to highlight the significant role of prenatal recognition of cord pathology, but also to issue a series of recommendations regarding therapeutic conduct in such situations. Full-term monitoring of the pregnant woman is a goal that was fulfilled in all the cases presented, given that no change in amniotic fluid volume, intrauterine growth restriction (IUGR), fetal Doppler parameters, or maternal comorbidities (hypertension, diabetes) was found. In the oligoamnios association or IUGR, careful fetal monitoring and personalized case management are required.

The role of the specialist in maternal and fetal medicine is to confirm or invalidate the suspicion of the true cord knot mentioned by the sonographer and to monitor high-risk pregnancies to term. Unfortunately, the reality shows that this pathology is most frequently diagnosed in tertiary centers.

## 5. Conclusions

In conclusion, a true umbilical cord knot can be easily visualized during second-trimester ultrasound screening for fetal anomalies, using the proposed algorithm that consists in systematic exploration by 2D imaging of umbilical cord between the insertion sites of each pregnancy, applying 3D-HD Flow Color Doppler or referring to a fetal-maternal center/specialist every umbilical cord entanglement that persists after fetal mobilization or two consecutive scans, and assessing umbilical artery flow by Doppler velocimetry. Pregnant women should be aware of the risk factors and about the rare possibility of intrauterine fetal death; they should be closely monitored in the third trimester, avoiding fetal hyperstimulation factors in the womb. The psychological factor of knowing the prenatal diagnosis of true umbilical knot is very important, so pregnant women need counseling to avoid iatrogenic prematurity. They should be explained the risks of preterm delivery, especially the consequences of respiratory distress syndrome. We consider that the infants should be delivered between 38 and 39 weeks of gestation by cesarean section because many studies report the installation of fetal bradycardia at the beginning of labor, and the incidence of vascular occlusion is very low before birth.

Furthermore, the analysis of umbilical artery flow velocimetry notch requires further investigation, as it can represent a sign of prognosis for the tightening of the knot or the cord entanglement in twin monoamniotic pregnancies.

### Practice Key Points

The strategy of prenatal diagnosis and monitoring true umbilical knots to term:

○ Assess umbilical cord free loops;
○ Verify the persistence of entanglement after fetal movement and repeated scan;
○ Use 3D-HD-flow imaging or refer the case for diagnosis confirmation;
○ Closely monitor umbilical artery flow by Doppler velocimetry.

## Figures and Tables

**Table 1:** Characteristics of the true umbilical cord knot patients diagnosed antenatally. SD—standard deviation.

Age, mean ± SD (years)	33 ± 5.06
Parity	
Primiparous	9
Secundiparous	5
Gestational age at diagnosis (weeks)	25.5 ± 6.72
Weight of newborns (grams)	3332.85 ± 277
Gestational age at birth	38.42 ± 1.15
Maternal anxiety	
Low	6
Moderate	3
High	5
Diabetic patients	4
Arterial hypertension	1
Fetal sex	
Male	5
Female	9
Amniotic fluid	
Normal	11
Polyhydramnios	2
Oligohydramnios	1
Nuchal cord	
No	7
One	4
Double	3

**Table 2:** The main studies investigating the link between umbilical cord knots and preterm birth.

Study	Year	Cases	Gestational Age at Birth (Weeks + Days)	Ultrasonographic Findings	Postnatal Examination	Obstetrical and Neonatal Outcomes
Hugon-Rodin [25]	2013	1 (twin pregnancy)	32	A clear notch was described on the first twin’s umbilical artery flow in a free cord loop at 31 weeks; TK undiagnosed prenatal	Tight TK found	Iatrogenic prematurity
Rodriguez [26]	2014	1	39	TK at 35^+5^ weeks	TK was confirmed	Elective cesarean with a favorable neonatal outcome
Aurioles-Garibay [27]	2014	2 (twin pregnancy)	32^+2^ (case 1) 32^+2^ (case 2)	Case 1: Cord entanglement umbilical artery notch in 1 twin at 26 weeks Case 2: Cord entanglement umbilical artery notch in both twins at 28 weeks	Case 1: Cord entanglement, forked placental cord insertion, and cord knot were confirmed Case 2: Cord entanglement and cord knot were confirmed	Case 1: Respiratory distress syndrome Case 2: Respiratory distress syndrome and hyperbilirubinemia
Polis [28]	2014	1	37	TK at 32 weeks	TK confirmed	Elective cesarean; Anxiety due to a previous intrauterine demise of a 37 weeks fetus with a true knot diagnosed postpartum
Ikechebelu [29]	2014	1	36	NA	TK confirmed	Neonatal death due to intrapartum asphyxia
Vasilj [30]	2015	1	39^+2^	TK at 27 weeks	TK was confirmed	Vaginal delivery with a favorable neonatal outcome
Bohiltea [31]	2016	133	36 (case 1) 36 (case 2) 36^+5^ (case 3) the rest at term	TK between 22–23 weeks in 16 cases (0.08% detection rate)	TK confirmed in all cases	Iatrogenic prematurity due to maternal anxiety (3 cases prenatally diagnosed) Prematurity of non-specified cause in 39 cases
da Cunha [17]	2016	1	30	IUGR at 25 weeks Placenta accreta TK at 29 weeks	TK confirmed	Emergency cesarean due to signs of brain sparing effect; Prematurity; IUGR
Zbeidy [32]	2017	1	36	IUGR at 36 weeks	TK and 4 NC confirmed	Iatrogenic prematurity for fetal distress; SGA
Sherer [33]	2017	3	36^+2^ (case 1) 39 (case 2) 36 (case 3)	Case 1: NC and TK at 36 weeks Case 2: NC and TK at 37 weeks Case 3: NC and TK at 29 weeks	Case 1: TK and NC confirmed Case 2: TK and NC confirmed Case 3: Two separate TK and NC confirmed	Case 1: emergency cesarean due to fetal bradycardia; prematurity Case 2: emergency cesarean due to fetal bradycardia. Case 3: emergency cesarean due to fetal bradycardia; prematurity.
Singh [18]	2020	1	37^+5^	A single loop of nuchal cord and true knot at 35 weeks	TK confirmed	Cesarean delivery on the mother’s request (anxiety)
Arrezo [34]	2020	1 (twin pregnancy)	32	NA	TK was diagnosed	Acute fetal distress
Weissmann-Brenner [35]	2021	867	<37	NA	TK confirmed	95 cases (10.95%) preterm deliveries

IUGR—intrauterine growth restriction, SGA—small for gestational age, NA—not applicable, TK—true knot, NC—nuchal cord; ^+5, +2^, represent the no of days (GA is expressed as weeks + days).

## Supplementary Materials

The following supporting information can be downloaded at: https://www.mdpi.com/article/10.3390/jcm11030818/s1, Video S1: Prenatal diagnosis of 7 cases of umbilical cord knot; the last one is a false umbilical knot. Table S1: Demographic for the patients.
