# Supplementary material for: The Strategy against Iatrogenic Prematurity Due to True Umbilical Knot: From Prenatal Diagnosis Challenges to the Favorable Fetal Outcome
Source: J Clin Med. 2022 Feb 3;11(3):818. doi: 10.3390/jcm11030818 (PMC8836486; doi:10.3390/jcm11030818)
Supplement: Supplementary file 1 [file jcm-11-00818-s001.zip › Supplementary-Table 2.pdf]

Table 2. The patients' characteristics from the group of 16 pregnancies were prenatally diagnosed with a true umbilical knot.

Cases	C1	C2	C3	C4	C5	C6	C7	C8	C9	C10	C11	C12	C13	C14	C15	C16
**GA at diagnosis**	16	22	30	22	22	16	24	22	24	22	37	37	30	23	30	32
**Maternal age**	32	28	28	30	31	30	34	34	28	35	31	31	34	41	42	42
**Fetal sex**	F	M	M	F	M	F	M	F	F	M	F	F	F	F	M	F
**Parity**	1	1	1	2	1	2	2	2	1	1	1	2	1	1	1	1
**Polyhydramnios**	No	No	No	No	Yes	No	No	No	No	No	No	Yes	No	oligoamnios	No	No
**Gestational diabetes mellitus**	No	Yes	No	No	No	No	No	Yes	No	Yes	Yes	No	No	Yes	No	No
**Pregnancy-induced hypertension**	No	No	No	No	No	No	No	No	No	Yes	No	No	No	No	No	Yes
**GA at birth**	38+3	39	39+1	38+2	38	38	39	38	37	37+6	41	39	38+3	38+5	39	36+4
**Weight at birth**	2950	3400	3500	3400	3800	2880	3650	3300	3000	3120	3600	3500	3290	3140	3400	3140
**Presence of nuchal cord**	No	No	Yes	Yes	Double	Double	Yes	No	No	Double	No	No	Yes	Double	No	Yes
**Level of maternal anxiety**	L	M	S	L	M	S	M	S	L	S	L	S	M	S	L	L
**Case particularity**	Double complex knot	-	-	-	Self-monitoring	Long cord	Self-monitoring	-	-	-	Meconial amniotic fluid	-	FIV	Double knot	-	FIV, 2700, M, velamentous insertion

M – male, F – female, L – low, M – moderate, S – severe.
